# Luciferase reporter mycobacteriophage (TM4::*GeNL*) enables rapid assessment of drug susceptibilities and inducible macrolide resistance in *Mycobacterium abscessus* complex

**DOI:** 10.1128/jcm.00841-25

**Published:** 2025-08-20

**Authors:** Saranathan Rajagopalan, Lahari Das, Donna J. Kohlerschmidt, Amy K. Rourke, Salika M. Shakir, Michelle H. Larsen, Max R. O’Donnell, Wendy A. Szymczak, Vincent E. Escuyer, Phyu M. Thwe, William R. Jacobs

**Affiliations:** 1 Department of Microbiology and Immunology, Albert Einstein College of Medicine 2006, New York, New York, USA; 2 New York State Department of Health, Wadsworth Center 116287, Albany, New York, USA; 3 Department of Pathology, The University of Utah School of Medicine 12348, Salt Lake City, Utah, USA; 4 ARUP Laboratories 33294 Salt Lake City, Utah, USA; 5 Division of Pulmonary, Allergy, and Critical Care Medicine, Columbia University Medical Center 21611, New York, New York, USA; 6 Department of Epidemiology, Mailman School of Public Health, Columbia University Medical Center 21611, New York, New York, USA; 7 Department of Pathology, Montefiore Medical Center 2013, New York, New York, USA; 8 Department of Pathology, Albert Einstein College of Medicine, Montefiore Medical Center 205134 , New York, New York, USA; The University of North Carolina at Chapel Hill School of Medicine, Chapel Hill, North Carolina, USA

**Keywords:** *Mycobacterium abscessus*, inducible macrolide resistance, amikacin, luciferase reporter mycobacteriophage, *erm(41)*

## Abstract

*Mycobacterium abscessus* (MAB) infections are challenging to treat due to high-level resistance to anti-tuberculosis drugs, carbapenems, fluoroquinolones, and tetracyclines. Clarithromycin and amikacin are considered cornerstone drugs for MAB treatment due to their superior efficacy; however, assessing clarithromycin susceptibility typically takes 7 to 14 days because of inducible macrolide resistance. We developed a 48 h luciferase reporter mycobacteriophage drug susceptibility testing (LRM-DST) assay using TM4::*GeNL* to evaluate MAB drug susceptibility. For this proof-of-principle study, we performed DST on 26 MAB clinical isolates using TM4::*GeNL* and Sensititre RAPMYCO2 plates. Genome sequencing was performed to identify the MAB sub-species and for understanding intrinsic and acquired drug resistance mechanisms. We detected clarithromycin resistance in 12/26 (46%) by both Sensititre and LRM-DST. All clarithromycin-resistant isolates carried full-length inducible erythromycin ribosomal methylase-41 [*erm(41*)] gene, and none had *rrl* (23S rRNA) mutations. Amikacin resistance or intermediate amikacin resistance was detected in 6/26 (23%) isolates. Mutations in the *rrs* (16S rRNA) gene at positions 1375A > G (*E. coli* 1408A > G) conferred amikacin resistance in four isolates; however, no mutations were identified in two of the amikacin intermediate-resistant isolates. LRM-DST results were in 100% and 92.3% concordance with Sensititre DST results of clarithromycin (kappa coefficient, 1.0; 95% confidence interval [CI], 0.84 to 1.0) and amikacin (kappa coefficient, 0.752; 95% confidence interval [CI], 0.43 to 1.0). In addition, LRM-DST results for bedaquiline, moxifloxacin, imipenem, linezolid, and cefoxitin correlated well with Sensititre DST. Our findings suggest that LRM-DST can provide reliable phenotypic MAB-DST information in 48 h for prompt clinical management.

**IMPORTANCE:**

*Mycobacterium abscessus* (MAB) is a notorious human pathogen causing severe infections in individuals with cystic fibrosis and immunocompromised patients. Treatment options for MAB are very limited as they are resistant to multiple drugs through intrinsic and acquired resistance mechanisms. While macrolides and amikacin are the key drugs in the fight against MAB infections, emerging drug resistance is compromising their efficacy. Current growth-based drug susceptibility testing (DST) method takes 7 to 14 days to identify clarithromycin susceptibility due to the presence of inducible macrolide resistance and 3 to 5 days for other drugs. We developed a novel luciferase reporter mycobacteriophage (LRM) based phenotypic DST method using TM4::*GeNL* to assess MAB drug susceptibility based on metabolic inhibition rather than growth. LRM-DST significantly reduces the DST turnaround time to 48 h and provides rapid assessment of MAB susceptibility to drugs, including inducible macrolide resistance, thereby accelerating the treatment process and improving patient outcomes.

## INTRODUCTION

Non-tuberculous mycobacteria (NTM) are ubiquitous in the environment and typically present as opportunistic pathogens in the setting of immunocompromised or structural lung disease (1). *Mycobacterium abscessus* (MAB), the most prevalent rapidly growing NTM, has garnered significant attention for its ability to cause pulmonary and extrapulmonary infections, predominantly in individuals with cystic fibrosis, chronic lung conditions, and immunocompromised patients (2–4). MAB infections are notoriously challenging to treat due to their intrinsic resistance towards first- and second-line anti-tuberculosis drugs, as well as carbapenems, fluoroquinolones, and tetracyclines (2, 4, 5). Drug resistance mechanisms in MAB include target modifications, acquired genes, permeability barriers, and overexpression of efflux pumps (6). Macrolides and amikacin (AMK), the bacteriostatic agents that inhibit protein synthesis by targeting the bacterial 50S and 30S ribosomal subunits, are the preferred drugs in the American Thoracic Society (ATS) recommended multi-drug regimen for treating MAB infections (6–8).

While macrolides such as clarithromycin (CLR) and azithromycin significantly improve outcomes and cure rates in drug-susceptible infections, their efficacy is often compromised by inducible resistance conferred by the erythromycin ribosomal methylase-41 [*erm(41*)] gene and mutations in the *rrl* gene encoding 23S rRNA (3, 9). Macrolide resistance due to inducible expression of the *erm(41*) gene is not detected until 7 to 14 days of conventional phenotypic drug susceptibility testing (DST) method; however, acquired resistance through mutations in the *rrl* gene can be detected as early as 3 days (10, 11). Hence, even if MAB exhibits macrolide susceptibility within the first 3 days of DST, an extended incubation up to 14 days is necessary to detect inducible macrolide resistance (10–14). Further, the most common mechanism of AMK resistance identified in MAB clinical isolates was through mutations in *rrs* (16S rRNA) gene at positions 1375 A > G (equivalent to *E. coli* 1408 A > G) (15). Broth microdilution is the only standard DST method recommended by the Clinical and Laboratory Standard Institute (CLSI) for MAB that provides minimal inhibitory concentration (MIC) by measuring bacterial growth in a series of drug dilutions (7, 10, 11, 13, 16). Some MAB isolates grow slowly in broth and may take up to 14 days to get sufficient growth for DST. Given the inherent instability of several MAB treatment drugs, CLSI M24 guidelines limit reliable reporting for slow-growing isolates after 5 days of incubation to only AMK and CLR (17).

Luciferase reporter mycobacteriophages (LRM) are engineered phages carrying a reporter gene, such as firefly luciferase, nanoluciferase, green enhanced nanoluciferase (*GeNL*), and fluorophores (18–21). LRMs infect and light up metabolically active mycobacteria and allow us to differentiate drug-susceptible and drug-resistant mycobacteria based on the luminescence readout (18, 19, 21, 22). The LRM-DST relies on the active metabolism rather than the replication of mycobacteria in the presence of drug, thereby shortening the turnaround time for DST results while providing highly accurate and reproducible susceptibility information (18, 19). Though LRMs have been previously demonstrated to facilitate rapid drug screening for *Mycobacterium leprae* and expedite DST turnaround time for *Mycobacterium tuberculosis* (MTB), their potential for NTM-DST including MAB remains unexplored (18, 19, 21–23). The recently engineered mycobacteriophage TM4::*GeNL* carrying Green-enhanced Nanoluciferase (*GeNL*) cassette has been shown to be the most sensitive LRM that enabled us to perform DST in *Mycobacterium tuberculosis* (MTB) clinical isolates in a 96-well format (18).

In this study, we present a novel approach using TM4::*GeNL* for rapid DST of MAB against AMK, CLR, cefoxitin (CEF), linezolid (LZD), moxifloxacin (MXF), imipenem (IMP), and bedaquiline (BDQ). LRM-DST significantly reduces the turnaround time for MAB DST to 48 h, which is critical for rapid clinical management of MAB infections. The retention of luciferase signal after drug treatment provides a readout value similar to conventional growth-based phenotypic DST in 48 h rather than up to 14 days and allows us to predict drug resistance.

## MATERIALS AND METHODS

### Strains, reagents, and media

All clinical isolates of MAB (*n* = 26) were retrieved from freezer stocks and cultured in Middlebrook 7H9 broth containing glycerol (0.2%), 10% OADC (oleic acid, albumin, dextrose, and catalase) and tyloxapol (0.05%). Only the first isolate from a patient was included in this study. *M. peregrinum* ATCC 700686 strain was used as a QC strain in both Sensititre and TM4::*GeNL* DST. All the drugs used in this study, such as AMK, CEF, CLR, IMP, LZD, MXF, and BDQ, were procured from MCE (MedChemExpress, USA).

### Phenotypic drug susceptibility testing

Phenotypic MIC testing was performed using Sensititre RAPMYCO2 Susceptibility Testing (ThermoFischer, USA) panel that includes AMK, CEF, CLR, IMP, LZD, MXF in Associated Regional and University Pathologists (ARUP) laboratories, Utah, USA. Following CLSI M24 guidelines, DST results were read after 72 h. If the clarithromycin DST result was recorded as susceptible (≤2 µg/mL) on day 3, the panel was read daily until day 14 to determine the presence of inducible macrolide resistance (17). All MIC values presented in this manuscript are determined according to CLSI M-07 guidelines (24). BDQ MIC testing was performed by standard broth microdilution method following standard protocol in our laboratory as it is not included in Sensititre RAPMYCO2 panel. BDQ drug dilutions (2×) were prepared in standard Middlebrook 7H9 media containing OADC, glycerol, and tyloxapol and dispensed (100 µL) into flat-bottom 96-well plates. Pre-cultured MAB clinical isolates (OD_600_ of ~0.8) were diluted to an OD_600_ 0.01, and 100  µL of the cell dilution was added to the plate to give a final OD_600_ of 0.005, and 1× drug concentration. The plates were incubated with gentle shaking, and bacterial growth was measured by OD_600_ after 72 h using a plate reader (Biotek Synergy, USA). MIC_90_ values were derived as the lowest drug concentration which had ≥ 90% growth reduction (OD_600_) compared with no-drug control.

### TM4::*GeNL* construction and optimization of drug susceptibility testing

Detailed methodology of the TM4::*GeNL* mycobacteriophage construction and purification has been described in our earlier study (18). Briefly, we amplified the *GeNL* cassette and assembled it into a plasmid that was subsequently ligated to the TM4 phage backbone (phAE159) and packaged to form shuttle phasmids. The resulting recombinant shuttle phasmid was electroporated into *Mycobacterium smegmatis* mc^2^155, and the plaques were picked, propagated, and purified. MAB cultures were grown to an OD_600_ of ~0.8, washed three times, and resuspended in the media lacking detergents. In 96-well white opaque plates, 50 µL of 2× dilution of drugs in Middlebrook 7H9 media containing OADC and glycerol was dispensed into each sample well, and 100 µL of media was added to the negative control wells. The no-drug control wells had 50 µL of media without drugs. Cultures were adjusted based on OD_600_ to have approximately 10^5^ cells per well and dispensed (50 µL) to designated no-drug control and drug-containing wells. Though MAB is a rapid grower with a doubling time of 4 to 5 h, the detection of critical inducible macrolide resistance could take up to 14 days by conventional methods. Hence, we investigated LRM-DST in 12 h (6 h of drug treatment followed by 6 h of phage infection), 24 h (12 h of drug treatment and 12 h of phage infection), and 48 h (24 h of drug treatment and 24 h of phage infection) formats. The plates were sealed and incubated at 37°C for 6 h/12 h/24 h followed by addition of TM4::*GeNL* phage (10^6^ PFU) in a volume of 10 µL to each sample and control wells. The plates were further incubated at 37°C for 6 h/12 h/24 h. Relative luminescence units (RLUs) were measured using Nano-Glo Luciferase Assay System (Promega, USA) following manufacturer’s instructions in a 96-well luminescence plate reader (Biotek Synergy, USA) at 12 h/ 24 h/48 h. The 48-h assay format was followed for LRM-DST based on our assay workflow optimization, which is schematically presented in Fig. S1. Drug dilutions with a reduction in RLU ≥ 90% compared with the no-drug control were considered as luminescence-based MIC_90_ cut-off driven by our previous study on optimization of TM4::*GeNL* DST in *M. tuberculosis* (18). This threshold proved highly effective by comparing luminescence retention after exposure to serial drug dilutions and achieved 100% concordance in classifying drug-susceptible and resistant clinical isolates. This cutoff-based classification was supported by both MGIT culture-based DST and whole-genome sequencing (WGS) data, while the drug-resistant isolates retained >10% RLU at the breakpoint concentration of each drug (18).

### Whole-genome sequencing and analysis

Genomic DNA from 26 MAB clinical isolates was isolated using CTAB manual extraction method (25). Quality and quantity of genomic DNA were checked using Nanodrop and Qubit (Invitrogen, USA). Whole-genome sequencing was performed by SeqCenter (Philadelphia, USA) using Illumina NovaSeq X Plus sequencer producing 2 × 151 bp paired-end reads. Demultiplexing, quality control, and adapter trimming were performed with bcl-convert1 (v4.2.4). *M. abscessus* subsp. *abscessus* strain GD20 (accession no. CP063327) was used as a reference to identify the *erm(41*) gene status, mutations in *rrl* (23S rRNA) and *rrs* (16S rRNA) genes. Trimmed reads were mapped to the genome using Bowtie2, and the SNPs were called using Geneious Prime (v2025.0.3) to identify the amino acid substitutions, deletions, and insertions. All the predicted drug resistance-associated mutations were validated using NTM-Profiler (https://bioinformatics.lshtm.ac.uk/ntm-profiler/).

### Statistical methods

The agreement between TM4::*GeNL* DST and Sensititre DST was assessed using Cohen’s kappa coefficient and concordance percentage, calculated with the *irr* statistical package in R (26). The breakpoint-based binary analyses to identify the difference in mean RLU signal retention between the Sensititre-confirmed susceptible and resistant isolates were assessed using two-sample, unpaired, Mann-Whitney *U* test (GraphPad Prism v10).

## RESULTS

### Conventional phenotypic DST results of *M. abscessus* complex clinical isolates

**Fig 1:**
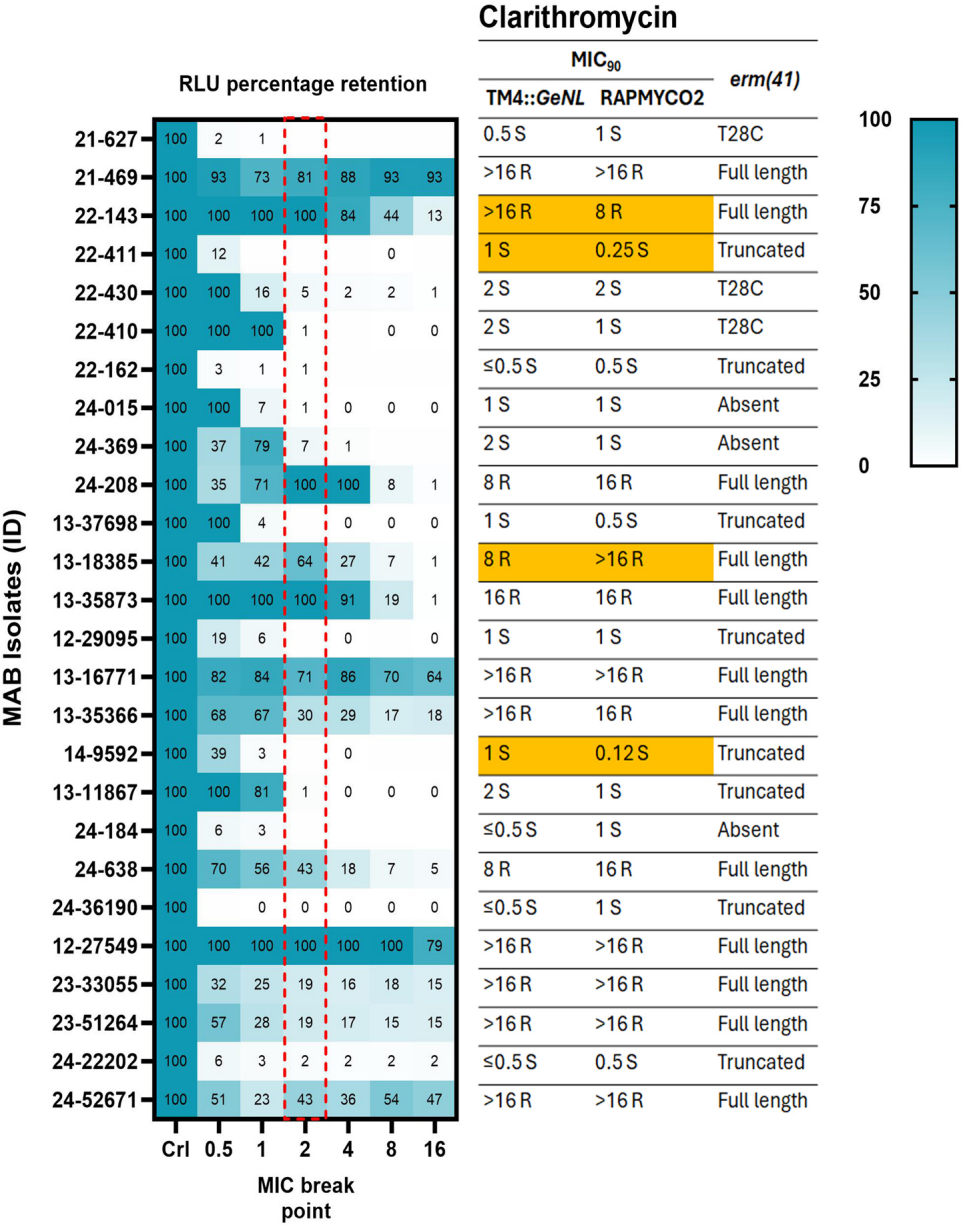
*M. abscessus* clarithromycin MIC_90_ values from TM4::*GeNL* DST, Sensititre RAPMYCO2 DST, and *erm (41)* gene status. This heatmap illustrates the signal retention in MAB clinical isolates (*n* = 26) infected with TM4::*GeNL* across different clarithromycin dilutions (0.5 to 16 µg/mL). The CLSI-recommended breakpoint (2 µg/mL) is highlighted with a red dotted box. For comparison, the corresponding MIC_90_ values for these MAB isolates, as determined by both TM4::*GeNL* DST and Sensititre DST, along with the *erm(41*) gene status, are shown in the right panel. Isolates with more than one dilution difference in MICs between the two methods are highlighted in orange.

**Fig 2:**
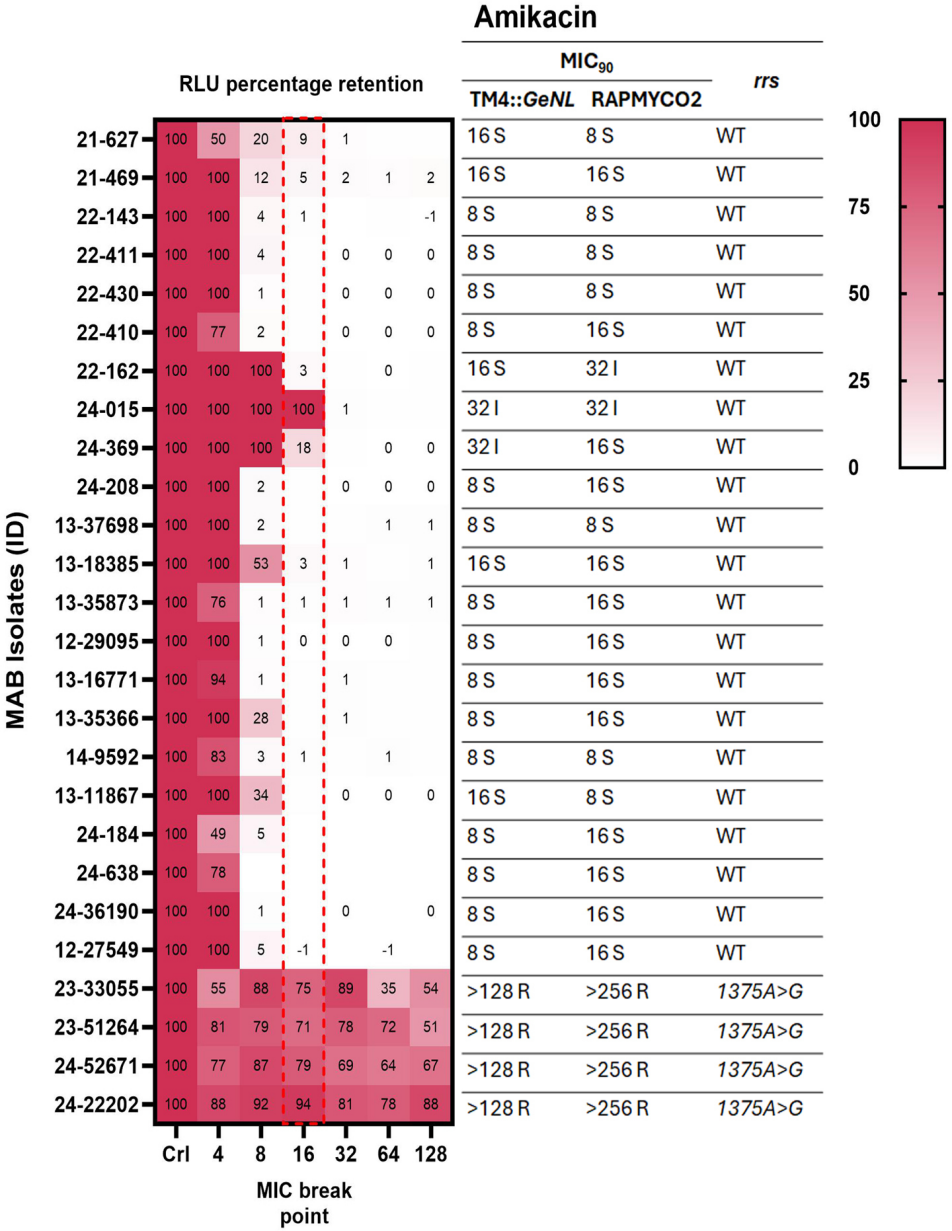
*M. abscessus* amikacin MIC_90_ values from TM4::*GeNL* DST, Sensititre RAPMYCO2 DST, and *rrs* gene mutations. This heatmap illustrates the signal retention in MAB clinical isolates (*n* = 26) infected with TM4::*GeNL* across different amikacin dilutions (4 to 128 µg/mL). The CLSI-recommended breakpoint (16 µg/mL) is highlighted with a red dotted box. For comparison, the corresponding MIC_90_ values for these MAB isolates, as determined by both TM4::*GeNL* DST and Sensititre DST, along with the *rrs* gene status, are shown in the right panel.

**Fig 3:**
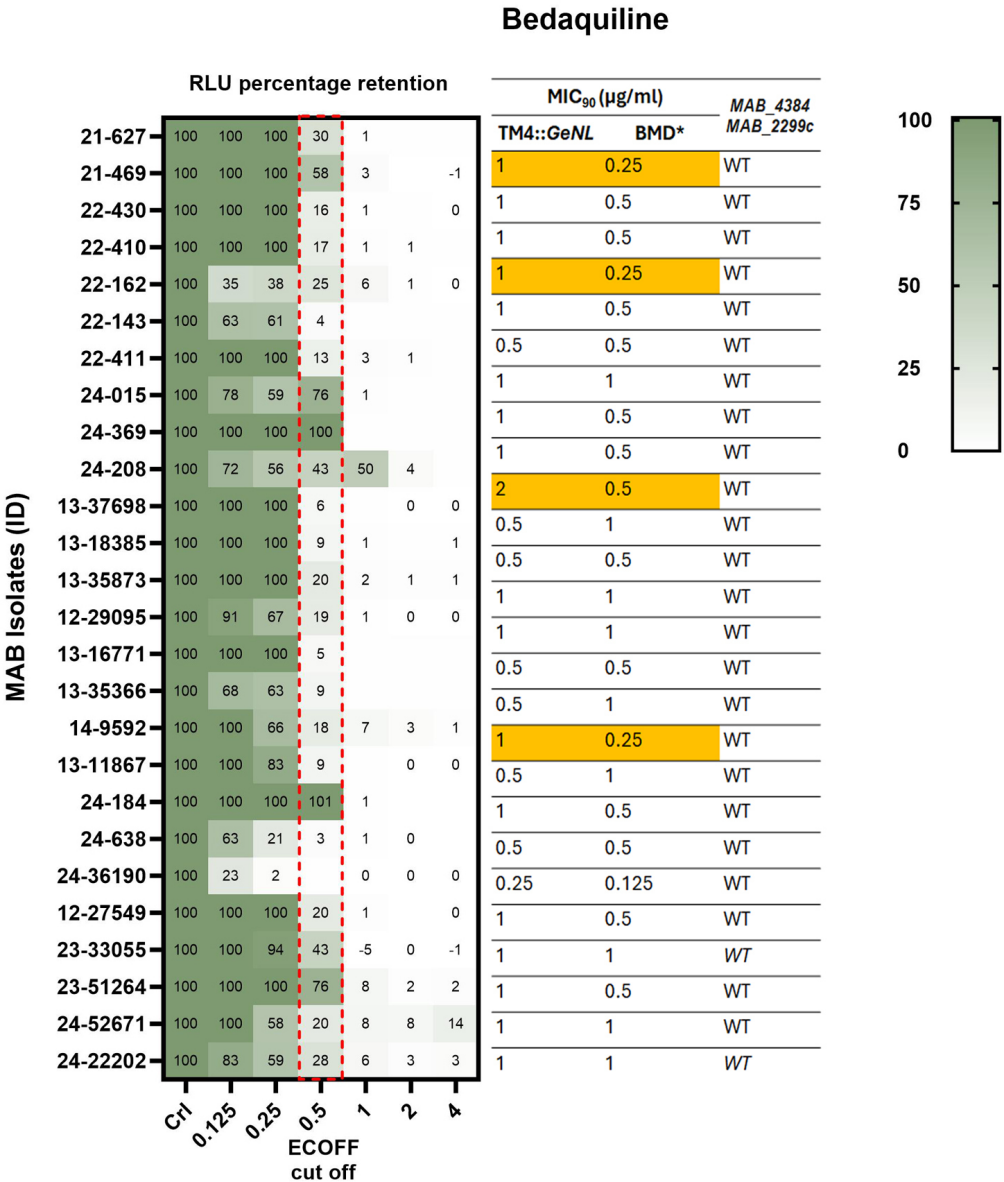
*M. abscessus* bedaquiline MIC_90_ values from TM4::*GeNL* DST, Sensititre RAPMYCO2 DST, *MAB_2299*c gene mutationsMAB_4384 and MAB_2299c genotypes. This heatmap illustrates the signal retention in MAB clinical isolates (*n* = 26) infected with TM4::*GeNL* across different bedaquiline concentrations (4 to 128 µg/mL). The Epidemiological Cut-Off (ECOFF) value from a recent meta-analysis (0.5 µg/mL) is highlighted with a red dotted box. Corresponding MIC_90_ values of the MAB isolates obtained by TM4::*GeNL* DST and Sensititre DST, along with the *MAB_2299*c and *MAB_4384* gene statuses, are shown on the right panel of the figure. Isolates with more than one dilution difference in MICs between the two methods are highlighted in orange. *BMD — Broth microdilution method, WT - Wild Type.

*M. abscessus* clinical isolates (*n* = 26) recovered from sputum, skin infections, and other specimens submitted to the clinical microbiology laboratory at Montefiore Medical Center, Bronx, NY and Wadsworth Center, the Public Health Reference Laboratory of the New York State Department of Health, were included in this study. We identified 12/26 (46%) isolates to be CLR resistant (MIC ≥ 8 µg/mL) by Sensititre DST (Table 1; Fig. 1). Interestingly, 11/12 (92%) CLR-resistant isolates were susceptible (MIC ≤ 2 µg/mL) on day 3 but became resistant (MIC ≥ 8 µg/mL) on the final reads at days 5 to 14, suggesting that they all had inducible macrolide resistance (Table 2; Fig. 2). AMK resistance (MIC > 64 µg/mL) was observed in 15% (4/26) of isolates, and two isolates showed intermediate resistance (MIC = 32 µg/mL) (Fig. 2). Interestingly, three of our isolates were resistant to both AMK and CLR, indicating co-resistance. Notably, all the isolates demonstrated either resistant (MIC ≥ 32 µg/mL) or intermediate (MIC = 8 or 16 µg/mL) MICs to IMP (Fig. S2), and MXF resistance (MIC ≥ 4) was observed in 26/26 isolates (Fig. S3). Furthermore, a high proportion of isolates showed reduced susceptibility to LZD and CEF, with 85% (22/26) having intermediate or resistant MICs (≥ 16 µg/mL) for LZD and 92% (24/26) exhibiting intermediate MIC (MIC = 32 or 64 µg/mL) for CEF (Fig. S4 and S5). Among the isolates tested, 35% (9/26) demonstrated BDQ MICs greater than 0.5 µg/mL, which is slightly higher than the epidemiological cut-off (ECOFF) from a MAB BDQ MIC meta-analysis (Fig. 3) (27).

**TABLE 1:** Agreement between Sensititre RAPMYCO2 DST and TM4::*GeNL* DST for clarithromycin and amikacin susceptibility testing^*a*^

Antibiotic (MIC breakpoint)	TM4::*GeNL* DST	Sensititre RAPMYCO2 DST		Overall concordance	Kappa coefficient (95% CI)	Fisher’s Exact *P*-value
		Susceptible	Resistant (intermediate)			
Clarithromycin (≥8 µg/mL)	Susceptible	14	0	100%	1 (0.84–1.0)	<0.0001
	Resistant	0	12			
Amikacin^*b*^ Resistant (≥128 µg/mL) Intermediate (32, 64 µg/mL)	Susceptible	20	(1)	92.3%	0.752 (0.43–1.0)	<0.0001
	Resistant (Intermediate)	(1)	4			

^*a*^ MIC – minimal inhibitory concentration; RAPMYCO2 – Sensititre susceptibility testing plate for rapidly growing mycobacteria; µg/mL – microgram/milliliter; TM4::*GeNL* – TM4 mycobacteriophage carrying Green-enhanced Nanoluciferase; DST – drug susceptibility testing. C.I. - class interval.

^*b*^ We combined the two-amikacin intermediate resistant isolates (MIC = 32 μg/mL) with resistant isolates for the purpose of Kappa coefficient analysis.

**TABLE 2:** Isolates with inducible macrolide resistance detected by TM4::*GeNL* at 48 h compared with MIC from extended incubation in Sensititre RAPMYCO2^*a*^

Sl no	Isolate ID	TM4::*GeNL*		RAPMYCO2	
		MIC (μg/mL)	Final Interpretation at 48 h	MIC (μg/mL)	Final Interpretation (Day reported)
1	21-469	>16	Resistant	>16	Resistant (Day 6)
2	21-143	>16	Resistant	8	Resistant (Day 6)
3	12-27549	>16	Resistant	>16	Resistant (Day 14)
4	13-16771	>16	Resistant	>16	Resistant (Day 14)
5	13-35366	>16	Resistant	8	Resistant (Day 14)
6	13-35873	16	Resistant	8	Resistant (Day 14)
7	13-18385	8	Resistant	16	Resistant (Day 14)
8	24-638	8	Resistant	16	Resistant (Day 8)
9	24-208	8	Resistant	8	Resistant (Day 3)
10	23-33055	>16	Resistant	>16	Resistant (Day 14)
11	23-51264	>16	Resistant	>16	Resistant (Day 14)
12	24-52671	>16	Resistant	>16	Resistant (Day 14)

^*a*^ MIC – minimal inhibitory concentration; µg/mL – microgram/milliliter; TM4::*GeNL* – TM4 mycobacteriophage carrying Green-enhanced Nanoluciferase; RAPMYCO2 – Sensititre susceptibility testing plate for rapidly growing mycobacteria.

### Rapid detection of clarithromycin (CLR) and amikacin (AMK) susceptibility using TM4::*GeNL* mycobacteriophage

**Fig 4:**
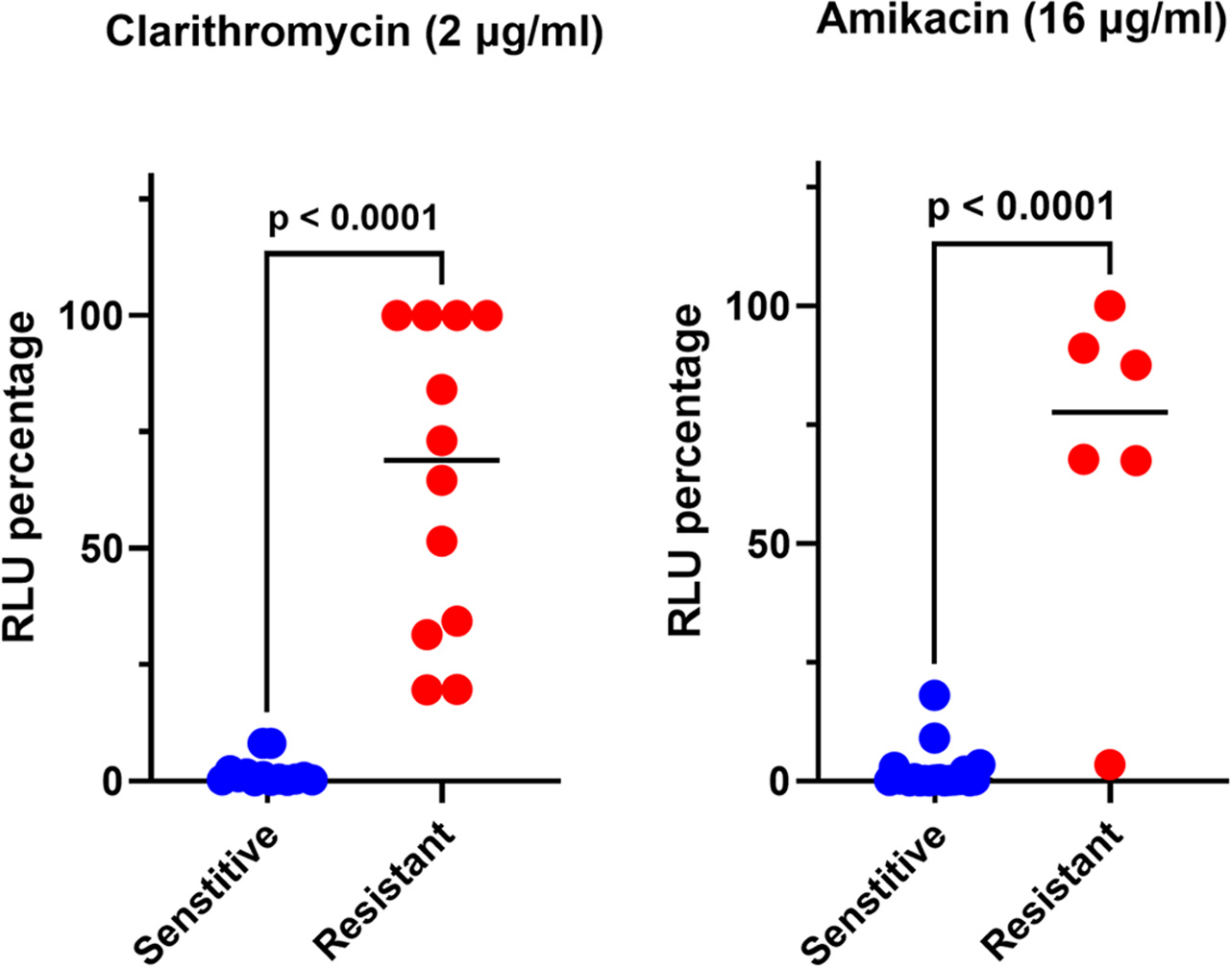
Luminescence retention among the MAB clinical isolates at breakpoint concentrations of clarithromycin and amikacin. Sensititre DST-confirmed susceptible and resistant isolates and their TM4::*GeNL* DST luminescence retention at the breakpoint concentration were plotted in this graph for CLR (**A**) and AMK (**B**) to demonstrate the assay’s adaptability to breakpoint-based binary DST analyses. The difference in signal retention was significant among the groups, and the statistical significance was assessed using the unpaired Mann-Whitney *U* test, and the *P*-values are mentioned in the graph. We combined the two amikacin intermediate-resistant isolates (MIC = 32 µg/mL) with resistant isolates for the purpose of breakpoint-based binary analysis.

We determined luminescence-based MIC_90_ of MAB clinical isolates using TM4::*GeNL*, defining it as the lowest concentration resulting in >90% reduction in signals compared to the no-drug control. We hypothesized that LRM-DST could offer a much faster turnaround for rapid-growing mycobacteria. We investigated CLR-DST in 12- and 24-h assay format, and we were unable to differentiate CLR susceptible and resistant isolates; a complete 48-h incubation was necessary for precise identification of CLR resistance using LRM-DST method (Fig. S6). LRM-DST identified 12/26 (46%) isolates to be CLR resistant based on luminescence retention (Fig. 1). We observed one- to eightfold variation in four isolates for CLR MICs; however, these differences did not affect the overall susceptibility interpretation of the isolates. There was 100% concordance (kappa coefficient, 1.0; 95% confidence interval [CI], 0.84 to 1.0) between the LRM-DST and Sensititre DST for detecting CLR resistance (Table 1). Furthermore, LRM-DST identified 11 isolates with inducible macrolide resistance in 48 h, which is significantly faster than the 7 to 14 days required by Sensititre DST (Table 2). Four AMK-resistant isolates (MIC > 256) were identified to be resistant by both LRM-DST and Sensititre DST (Fig. 2). Among the two isolates with an intermediate AMK MIC (MIC = 32 µg/mL) according to phenotypic DST, one was classified as susceptible (MIC = 16 µg/mL) by LRM-DST. Conversely, a second isolate was AMK susceptible (MIC = 16 µg/mL) in Sensititre DST and showed intermediate resistance (MIC = 32 µg/mL) in LRM-DST. Despite the single dilution difference between the methods, these two isolates had different phenotypes as the MIC differences were at the breakpoint concentration. We observed 92.3% concordance (kappa coefficient, 0.752; 95% confidence interval [CI], 0.43 to 1.0) between the methods for detecting AMK resistance (Table 1). We further analyzed the results using breakpoint-based binary analyses to demonstrate the assay’s adaptability for both MIC_90_ and binary (susceptible/resistant) reporting. We observed significant differences (*P* < 0.0001) in signal retention among the CLR and AMK susceptible and resistant isolates at their breakpoint concentration (CLR: 2 µg/mL; AMK: 16 µg/mL) (Fig. 4A and B). Due to the small sample size, we combined the two amikacin intermediate-resistant isolates and categorized them with resistant isolates for the purpose of Kappa coefficient calculation and breakpoint-based binary analysis (Table 1 and Fig. 4A and B).

### Genetic mechanisms of clarithromycin and amikacin resistance

We performed Illumina shotgun sequencing of all MAB clinical isolates and the median coverage depth ranged from 74× to 385×. Subspecies identification using NTM-Profiler revealed 13 as *M. abscessus* subsp. *abscessus*, 11 as *M. abscessus* subsp. *massiliense,* and two as *M. abscessus* subsp. *bolletti* isolates (Table S1). All 12 (100%) CLR-resistant isolates (MIC ≥ 8 µg/mL) had intact full-length *erm(41*), and none of them had *rrl* mutations conferring intrinsic resistance (Fig. 1). Among the CLR-susceptible (MIC ≤ 2 µg/mL) isolates, 11/14 (79%) were *M. abscessus* subsp. *massiliense* and had the non-functional *erm(41*) with a 274 bp deletion, 3/14 (21%) had *erm(41*) T28C substitution, which is a loss of function mutation (Fig. 1). All four AMK-resistant (MIC > 64 µg/mL) isolates had mutations in *rrs* at position 1375A > G (*E. coli* numbering A1408G), and no other additional mutations were identified (Fig. 2).

We analyzed *gyrA* and *gyrB* sequences from our WGS data, and we could not identify any known mutations that could confer MXF resistance, suggesting that the resistance might be contributed by mechanisms other than target modifications. Also, we could not associate LZD, CEF, and IMP resistance with any target or acquired genes. While the precise mechanisms of MAB resistance to LZD, CEF, and IMP remain unclear, drug efflux and permeability barriers could be the most probable contributing factors (6). We found no mutations in the MAB_2299c and MAB_4384 sequences (both *mmpR* homologs in MAB) that were unique to isolates with elevated BDQ MICs (28).

### LRM-DST of bedaquiline, imipenem, moxifloxacin, cefoxitin, and linezolid

Despite its usage as a salvage treatment in limited cases of MAB infections, BDQ has shown significant potential for improving patient symptoms (27). We performed LRM-DST using TM4::*GeNL* on MAB clinical isolates to determine BDQ MICs and compared them with broth microdilution DST results. We observed identical MICs in 10 out of 26 isolates (0.5 or 1 µg/mL), a one-dilution difference in 12 isolates, and a four-dilution difference in four isolates (Fig. 3). Given the absence of established BDQ MIC guidelines for MAB, we relied on the ECOFF value, which is the highest MIC value within a population from a prior meta-analysis and identified 18/26 (69%) had higher MICs (Fig. 3) than ECOFF (27). All isolates exhibited intermediate resistance or resistance to IMP (MIC ≥ 8 µg/mL) and CEF (MIC ≥ 32 µg/mL), 25/26 (96%) were resistant to MXF (MIC ≥ 2 µg/mL) (Fig. S2 to S4). Notably, LRM-DST indicated resistance to LZD (≥ 64 µg/mL) in 25/26 (96%) isolates, whereas Sensititre identified three of those resistant isolates as susceptible (Fig. S5). Furthermore, five isolates showed greater than one-dilution difference in LZD MICs between the two DST methods (Fig. S5).

## DISCUSSION

In the United States, the annual incidence of NTM lung disease rose from 6.78 per 100,000 persons in 2008 to 11.70 per 100,000 persons in 2015, indicating a clear upward trend (29). Among the rapidly growing mycobacteria (RGM), MAB infections are extremely difficult to treat due to the presence of intrinsic and acquired resistance towards multiple classes of antibiotics (2, 5, 11). Though MAB is a rapidly growing mycobacteria, it takes up to 3 to 5 days for most DST results and up to 14 days to get macrolide DST results due to the presence of intrinsic inducible macrolide resistance encoded by the *erm(41*) gene (7, 14). MAB complex comprises three subspecies: *M. abscessus* subsp. *abscessus, M. abscessus* subsp. *bolletii,* and *M. abscessus* subsp. *massiliense,* among which *massiliense* is known to be susceptible to macrolides due to the truncated *erm (41*) gene (30, 31). Susceptibility of *M. abscessus* subsp. *abscessus* and *M. abscessus* subsp. *bolletii* to macrolides is determined by the *erm(41*) sequevar, specifically the nucleotide at base position 28. The C28 sequevar is susceptible, while the T28 sequevar is resistant (30).

DNA-based molecular diagnostic assays, including NTM-DR and high-resolution melting curve analysis, exhibit high sensitivity and specificity (>96%); however, they are limited to CLR and AMK, detect known drug-resistant mutations, and are unable to identify variants outside the mutation hotspot (32, 33). MALDI-TOF MS-based methods showed sub-optimal performance in detecting CLR resistance, with a sensitivity and a specificity of 82% and 57% (34). Nevertheless, phenotypic DST remains the gold standard for MAB DST despite the lengthy process and the need for specialized technologists (7, 11, 16). Hence, a rapid phenotypic approach would be an ideal alternative to shorten the turnaround time and thereby expedite the treatment process.

The LRM-DST format is simple and similar to the Sensititre plate DST, requiring an incubator and a plate reader to measure the luminescence readout (18, 19). The rapid 48-h timeframe for LRM-DST to determine inducible macrolide resistance is primarily due to the assay’s underlying mechanism which measures MAB viability through Green enhanced nanoluciferase expression driven by active metabolism (18). While we observed an initial increase in luminescence signal at 24-h assay format, inducible CLR resistance was detected only at 48 h (Fig. S6). In an earlier study, Nash et al. showed that *erm(41*) expression increased by 50-fold as early as 9 h following CLR exposure in MAB (35). This finding corroborates our observation that even initial *erm(41*) levels are sufficient to maintain bacterial viability, a phenomenon precisely captured by our assay (35). By 48 h, the induction of *erm(41*) and subsequent ribosomal modification likely falls below the threshold needed to manifest discernible growth (35). However, our LRM-DST method is sensitive enough to capture this initial onset, enabling clear differentiation of strains with active inducible macrolide resistance. Hence, we followed this format and conducted a proof of principle study to assess the performance of LRM-DST for CLR, AMK, MXF, LZD, IMP, CEF, and BDQ. Among the 26 clinical isolates tested, we obtained 100% concordance for CLR and 92.3% concordance for AMK compared with Sensititre DST. We have also demonstrated the assay’s adaptability to breakpoint-based binary DST (Fig. 4A and B). Since CLR susceptibility is a strong predictor of treatment outcome among MAB infections, an increasing trend of CLR resistance, including inducible resistance, is alarming, and early identification is empirical for treating MAB infections (36, 37).

The LRM-DST results of MXF, IMP, CEF, LZD, and BDQ showed a strong correlation with Sensititre results, with values being either identical or differing by one to four dilutions. Such differences between our quantitative luminescence-based phage MIC and the visually assessed Sensititre MIC are expected given their distinct methodological approaches (18, 19). It is reasonable to assume that a bacterial population can remain metabolically active even when its growth is inhibited, which could explain the observed differences. In addition, static drugs, such as LZD, can exhibit trailing end points, which indicate reduced but persistent growth at higher drug concentrations, causing variations in MIC interpretation among clinical labs, and it is a well-known challenge for LZD susceptibility testing (38). Since LRM-DST provides luminescence signals when it encounters metabolically active bacteria, it is likely that the trailing points could have metabolically active viable MAB.

Overall, we have demonstrated the ability of the LRM-DST method to precisely determine the AMK, CLR, and other drug susceptibilities in clinical MAB isolates. Our DST approach for MAB is novel and would potentially shift the current paradigm of MAB DST in clinical labs. Though our results provide compelling evidence for LRM-DST having promising potential, the limitation of this study is the low sample size of clinical isolates. Our ultimate goal is to expand LRM-DST to the clinical settings through multicentric studies for the performance evaluation and feasibility of incorporating this in the clinical laboratory workflow. In summary, LRM-DST offers an efficient and rapid method to assess MAB sensitivity to drugs irrespective of their mode of action and reduces the MAB DST turnaround time from up to 14 days to 48 h. Since it is a phenotypic approach, it can be expanded to all existing and new drugs, including bedaquiline, which is currently being used as a salvage treatment option for multidrug-resistant MAB.

## Data Availability

The whole genome sequence read files of all the MAB clinical isolates were deposited in SRA database under the BioProject ID PRJNA1261920. A complete list of Isolate IDs sequenced in this study and their SRA accession numbers (SRR33530883 to SRR33530908) are given in Table S1.

## SUPPLEMENTAL MATERIAL

Supplemental tables and figures jcm.00841-25-s0001.docx Table S1 and Fig. S1 to S6.

ASM does not own the copyrights to Supplemental Material that may be linked to, or accessed through, an article. The authors have granted ASM a non-exclusive, world-wide license to publish the Supplemental Material files. Please contact the corresponding author directly for reuse.
