# Supplementary material for: Luciferase reporter mycobacteriophage (TM4::GeNL) enables rapid assessment of drug susceptibilities and inducible macrolide resistance in Mycobacterium abscessus complex
Source: J Clin Microbiol. 2025 Aug 20;63(9):e00841-25. doi: 10.1128/jcm.00841-25 (PMC12421814; doi:10.1128/jcm.00841-25)

***Supplementary files***

***__________________________________________________________________***

**Luciferase Reporter Mycobacteriophage (TM4::*GeNL*) enables rapid assessment of drug susceptibilities and inducible macrolide resistance in *Mycobacterium abscessus* complex**

Saranathan Rajagopalan^1^, Lahari Das^1^, Donna J Kohlerschmidt^2^, Amy K Rourke^2^, Salika M Shakir^3,4^, Michelle H Larsen^1^, Max R O’Donnell^5,6^, Wendy A Szymczak^7,8^, Vincent E Escuyer^2^, Phyu M Thwe^7,8*^ & William R Jacobs Jr.^1*^

^1^Department of Microbiology and Immunology, Albert Einstein College of Medicine, Bronx, New York, USA.

^2^Wadsworth Center, New York State Department of Health, Albany, New York, USA.

^3^Department of Pathology, University of Utah School of Medicine, Salt Lake City, Utah, USA

^4^ARUP Laboratories, Salt Lake City, Utah, USA

^5^Division of Pulmonary, Allergy, and Critical Care Medicine, Columbia University Medical Center, New York, New York, USA.

^6^Department of Epidemiology, Mailman School of Public Health, Columbia University Medical Center, New York, New York, USA.

^7^Department of Pathology, Montefiore Medical Center, Bronx, New York, USA.

^8^Department of Pathology, Albert Einstein College of Medicine, Montefiore Medical Center, Bronx, New York, USA

***Equal contribution as corresponding author:**

William R. Jacobs, Jr., PhD, Albert Einstein College of Medicine, 1301 Morris Park Avenue, Bronx, New York 10461, USA. Phone: +1 (718) 678-1075; Fax: +1 (718) 678-1022.

Email: william.jacobs@einsteinmed.edu

Phyu M Thwe, Ph.D., Montefiore Medical Center, 111 E 210th Street, Bronx. NY 10467.

Ph: 718-920-4189; Fax: 718-654-7402.

Email: pthwe@montefiore.org

**Table S1. Whole genome sequencing accession numbers of *M. abscessus* clinical isolates. BioProject PRJNA1261920**

| **Accession** | **Isolate ID** | **Organism** |
| --- | --- | --- |
| SAMN48440878 | 21-627 | *Mycobacterium abscessus* subsp*. abscessus* |
| SAMN48440879 | 21-469 | *Mycobacterium abscessus* subsp*. abscessus* |
| SAMN48440880 | 22-143 | *Mycobacterium abscessus* subsp*. bolletii* |
| SAMN48440881 | 22-411 | *Mycobacterium abscessus* subsp*. massiliense* |
| SAMN48440882 | 22-430 | *Mycobacterium abscessus* subsp*. abscessus* |
| SAMN48440883 | 22-410 | *Mycobacterium abscessus* subsp*. abscessus* |
| SAMN48440884 | 22-162 | *Mycobacterium abscessus* subsp*. massiliense* |
| SAMN48440885 | 12-27549 | *Mycobacterium abscessus* subsp*. bolletii* |
| SAMN48440886 | 12-29095 | *Mycobacterium abscessus* subsp*. massiliense* |
| SAMN48440887 | 13-11867 | *Mycobacterium abscessus* subsp*. massiliense* |
| SAMN48440888 | 13-16771 | *Mycobacterium abscessus* subsp*. abscessus* |
| SAMN48440889 | 14-9592 | *Mycobacterium abscessus* subsp*. massiliense* |
| SAMN48440890 | 13-35366 | *Mycobacterium abscessus* subsp*. abscessus* |
| SAMN48440891 | 13-35873 | *Mycobacterium abscessus* subsp*. abscessus* |
| SAMN48440892 | 13-37698 | *Mycobacterium abscessus* subsp*. massiliense* |
| SAMN48440893 | 13-18385 | *Mycobacterium abscessus* subsp*. abscessus* |
| SAMN48440894 | 24-36190 | *Mycobacterium abscessus* subsp*. massiliense* |
| SAMN48440895 | 24-184 | *Mycobacterium abscessus* subsp*. massiliense* |
| SAMN48440896 | 24-638 | *Mycobacterium abscessus* subsp*. abscessus* |
| SAMN48440897 | 24-015 | *Mycobacterium abscessus* subsp*. massiliense* |
| SAMN48440898 | 24-369 | *Mycobacterium abscessus* subsp*. massiliense* |
| SAMN48440899 | 24-208 | *Mycobacterium abscessus* subsp*. abscessus* |
| SAMN48440900 | 23-33055 | *Mycobacterium abscessus* subsp*. abscessus* |
| SAMN48440901 | 23-51264 | *Mycobacterium abscessus* subsp*. abscessus* |
| SAMN48440902 | 24-22202 | *Mycobacterium abscessus* subsp*. massiliense* |
| SAMN48440903 | 24-52671 | *Mycobacterium abscessus* subsp*. abscessus* |

**Supplementary figures legends**

**Figure S1. Schematic workflow of the LRM DST (Luciferase Reporter Mycobacteriophage Drug Susceptibility Testing).** Clinical isolates of *Mycobacterium abscessus* were cultured and washed in Middlebrook 7H9 media. Fifty microliters of the bacterial suspension were then inoculated into the wells of a white opaque 96-well plate, each containing 50 µl of antimicrobial agents at a 2X working concentration. Plates were incubated at 37°C for 24 hours. Following this initial incubation, TM4::*GeNL* phage was added to each well, and the plates were incubated for an additional 24 hours. After a total incubation period of 48 hours, the NanoGlo luciferase assay system substrate (Promega, USA) was dispensed into each well. Luminescence signals were subsequently measured and analyzed.

**Figure S2. *M. abscessus* imipenem MIC_90_ values from TM4::*GeNL* DST and Sensititre RAPMYCO2 DST.** Percentage of signal retention in MAB clinical isolates (*n*=26) infected with TM4::*GeNL* across different imipenem dilutions (0.25 to 8 µg/ml). The CLSI-recommended breakpoint (4 µg/ml) is highlighted with a red dotted box. For comparison, the corresponding MIC_90_ values for these MAB isolates, as determined by both TM4::*GeNL* DST and Sensititre DST, are shown in the right panel.

**Figure S3. *M. abscessus* moxifloxacin MIC_90_ values from TM4::*GeNL* DST and Sensititre RAPMYCO2 DST.** Percentage of signal retention in MAB clinical isolates (*n*=26) infected with TM4::*GeNL* across different moxifloxacin dilutions (0.25 to 8 µg/ml). The CLSI-recommended breakpoint (1 µg/ml) is highlighted with a red dotted box. For comparison, the corresponding MIC_90_ values for these MAB isolates, as determined by both TM4::*GeNL* DST and Sensititre DST, are shown in the right panel. Isolates with discrepant sensitivity results between the two methods are highlighted in orange.

**Figure S4. *M. abscessus* cefoxitin MIC_90_ values from TM4::*GeNL* DST and Sensititre RAPMYCO2 DST.** Percentage of signal retention in MAB clinical isolates (*n*=26) infected with TM4::*GeNL* across different cefoxitin dilutions (4 to 128 µg/ml). The CLSI-recommended breakpoint (16 µg/ml) is highlighted with a red dotted box. For comparison, the corresponding MIC_90_ values for these MAB isolates, as determined by both TM4::*GeNL* DST and Sensititre DST, are shown in the right panel.

**Figure S5. *M. abscessus* linezolid MIC_90_ values from TM4::*GeNL* DST and Sensititre RAPMYCO2 DST.** Percentage of signal retention in MAB clinical isolates (*n*=26) infected with TM4::*GeNL* across different cefoxitin dilutions (2 to 64 µg/ml). The CLSI-recommended breakpoint (8 µg/ml) is highlighted with a red dotted box. For comparison, the corresponding MIC_90_ values for these MAB isolates, as determined by both TM4::*GeNL* DST and Sensititre DST, are shown in the right panel. Isolates with discrepant sensitivity results between the two methods are highlighted in orange.

**Figure S6. Optimization of *M. abscessus* LRM-DST for clarithromycin (CLR) using TM4::*GeNL*.** We performed CLR DST using TM4::*GeNL,* testing various time formats including 12-hour, 24-hour, and 48-hour. A full 48-hour format (comprising 24 hours of drug treatment followed by 24 hours of phage infection) proved essential. This extended incubation was necessary to both identify inducible macrolide resistance and obtain an MIC value comparable to those from Sensititre DST. Isolates with more than one dilution difference in MICs at different timepoints between the two methods are highlighted in orange.

**Figure S1**
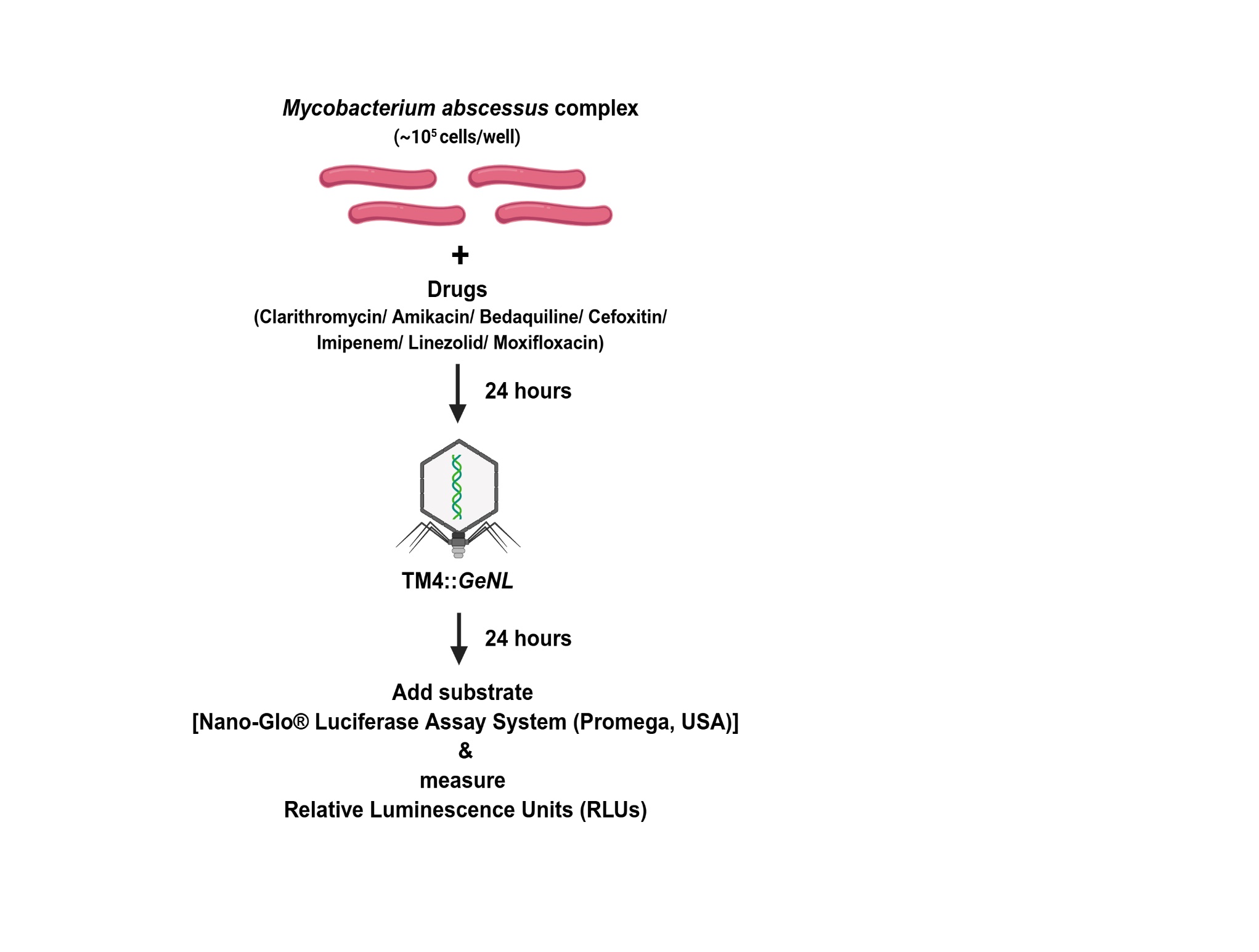
**. Schematic workflow of the LRM DST (Luciferase Reporter Mycobacteriophage – Drug Susceptibility Testing).**

**Figure S2. *M. abscessus* imipenem MIC_90_ values from TM4::*GeNL* DST and Sensititre^TM^ RAPMYCO2 DST**


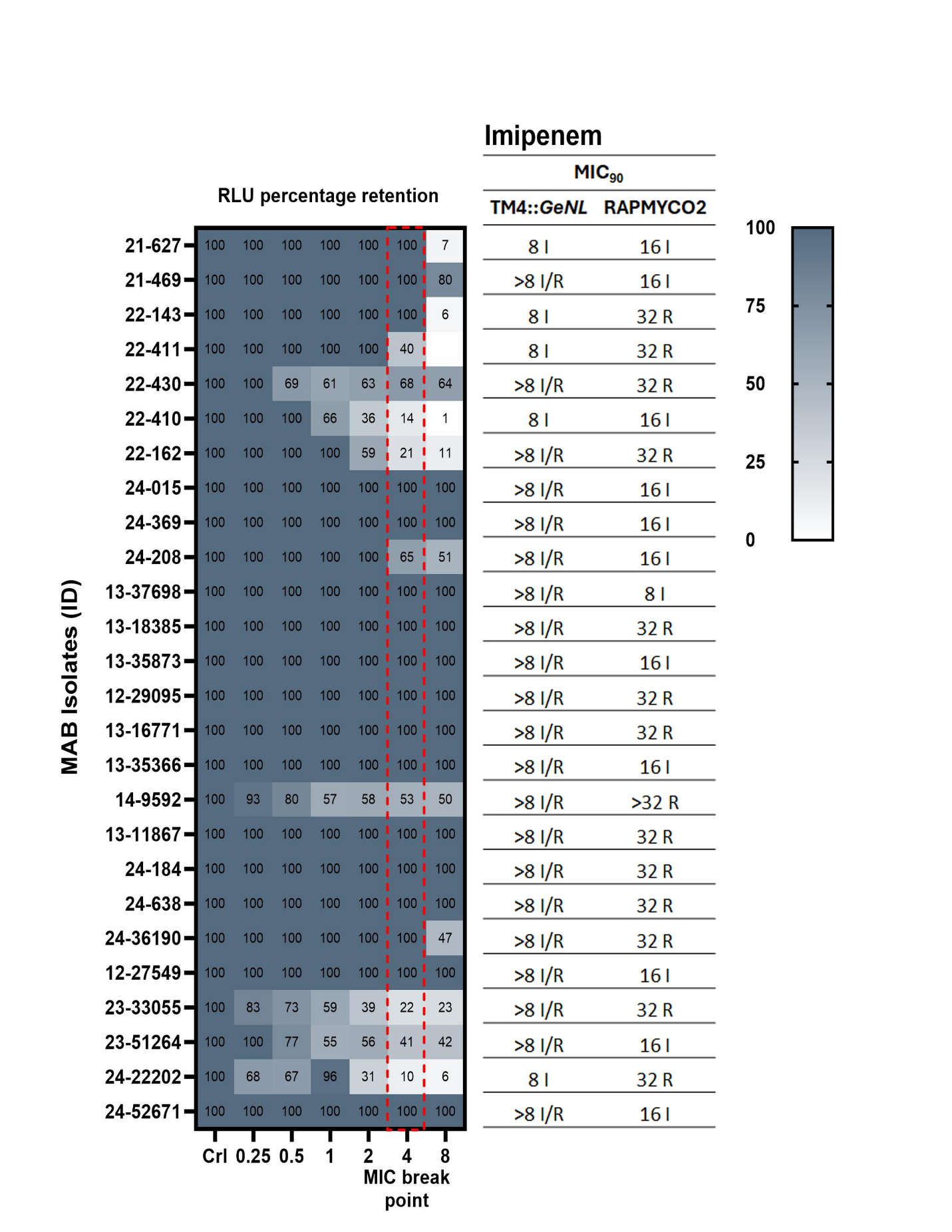


**Figure S3. *M. abscessus* moxifloxacin MIC_90_ values from TM4::*GeNL* DST and Sensititre^TM^ RAPMYCO2 DST**


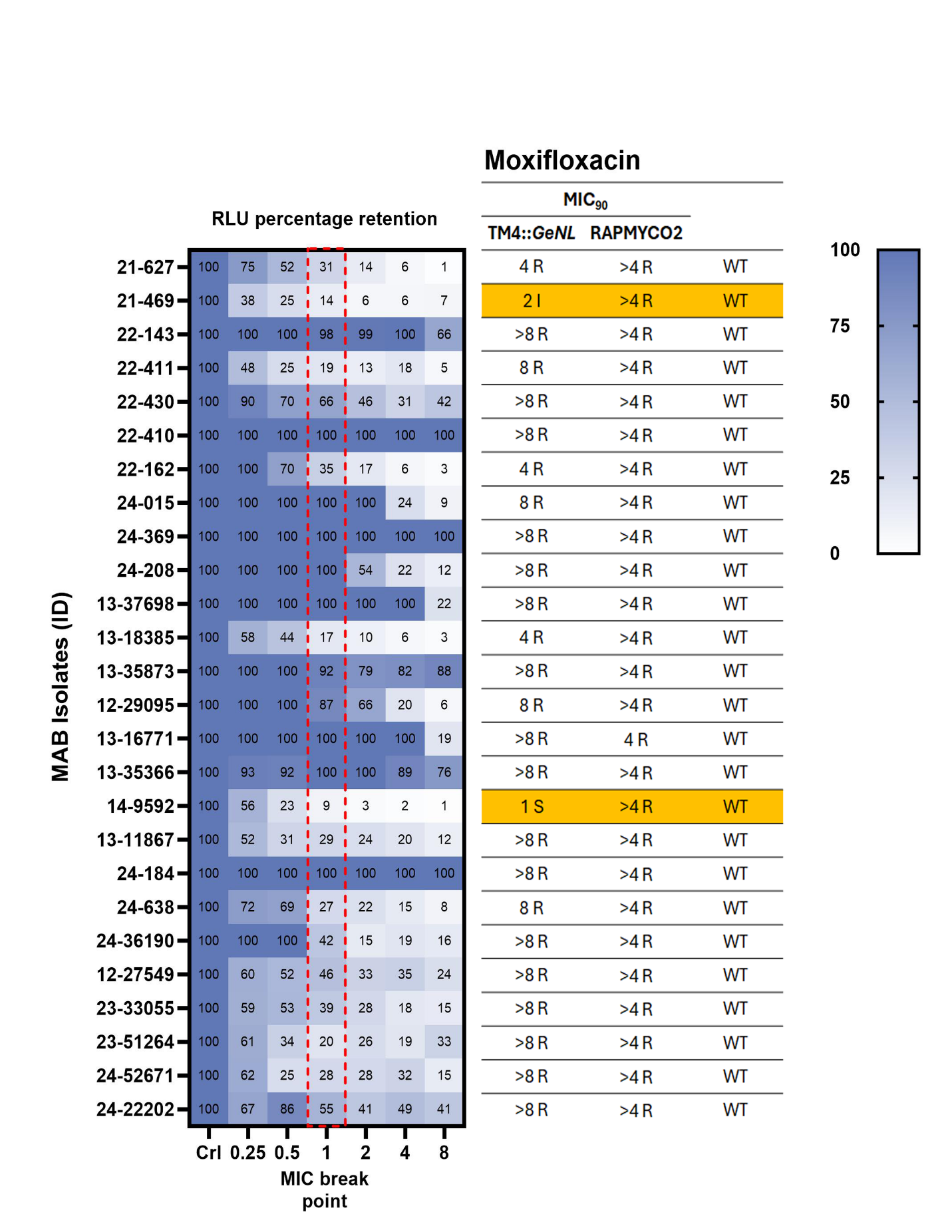
**Figure S4. *M. abscessus* cefoxitin MIC_90_ values from TM4::*GeNL* DST and Sensititre^TM^ RAPMYCO2 DST**


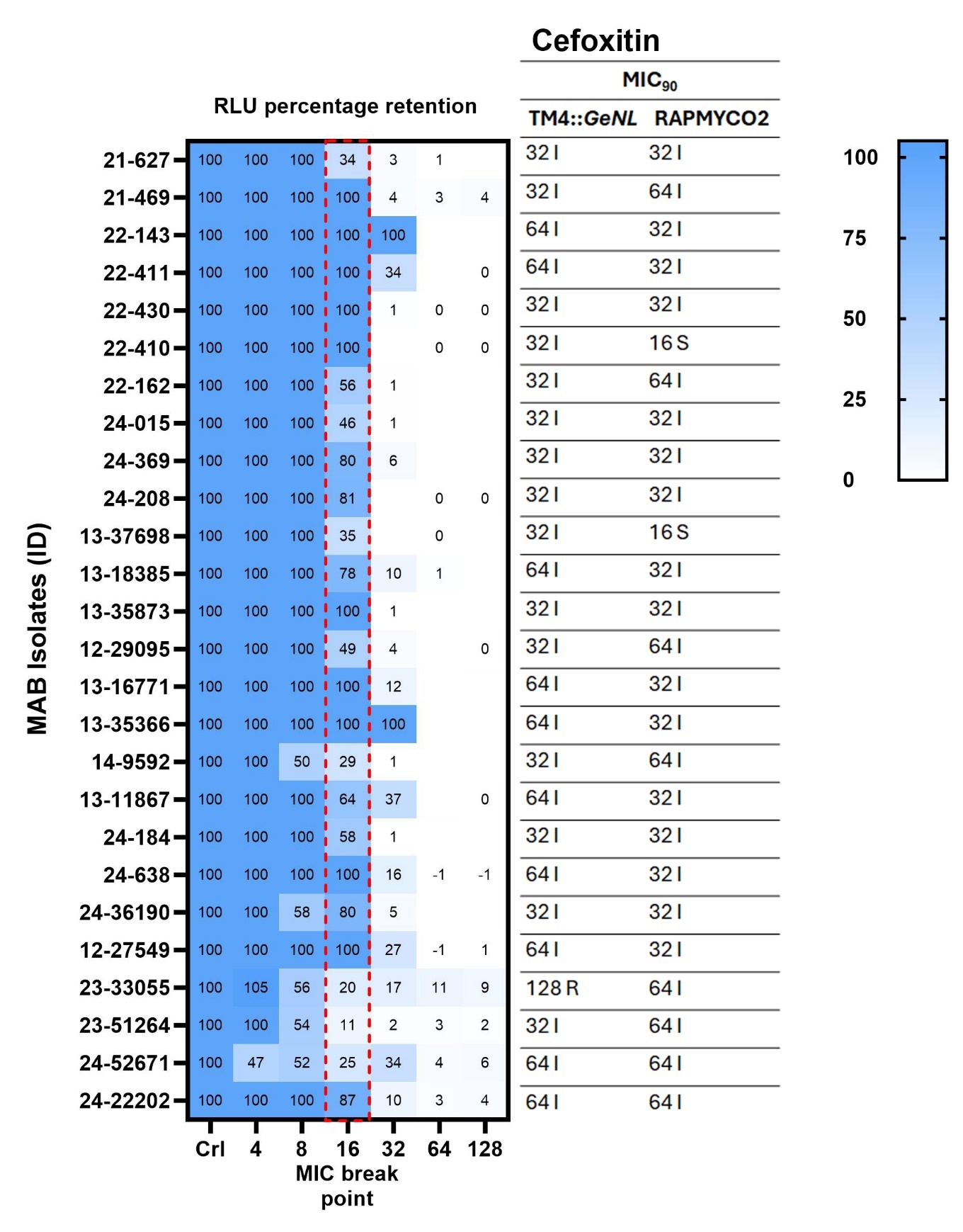


**Figure S5. *M. abscessus* linezolid MIC_90_ values from TM4::*GeNL* DST and Sensititre^TM^ RAPMYCO2 DST**


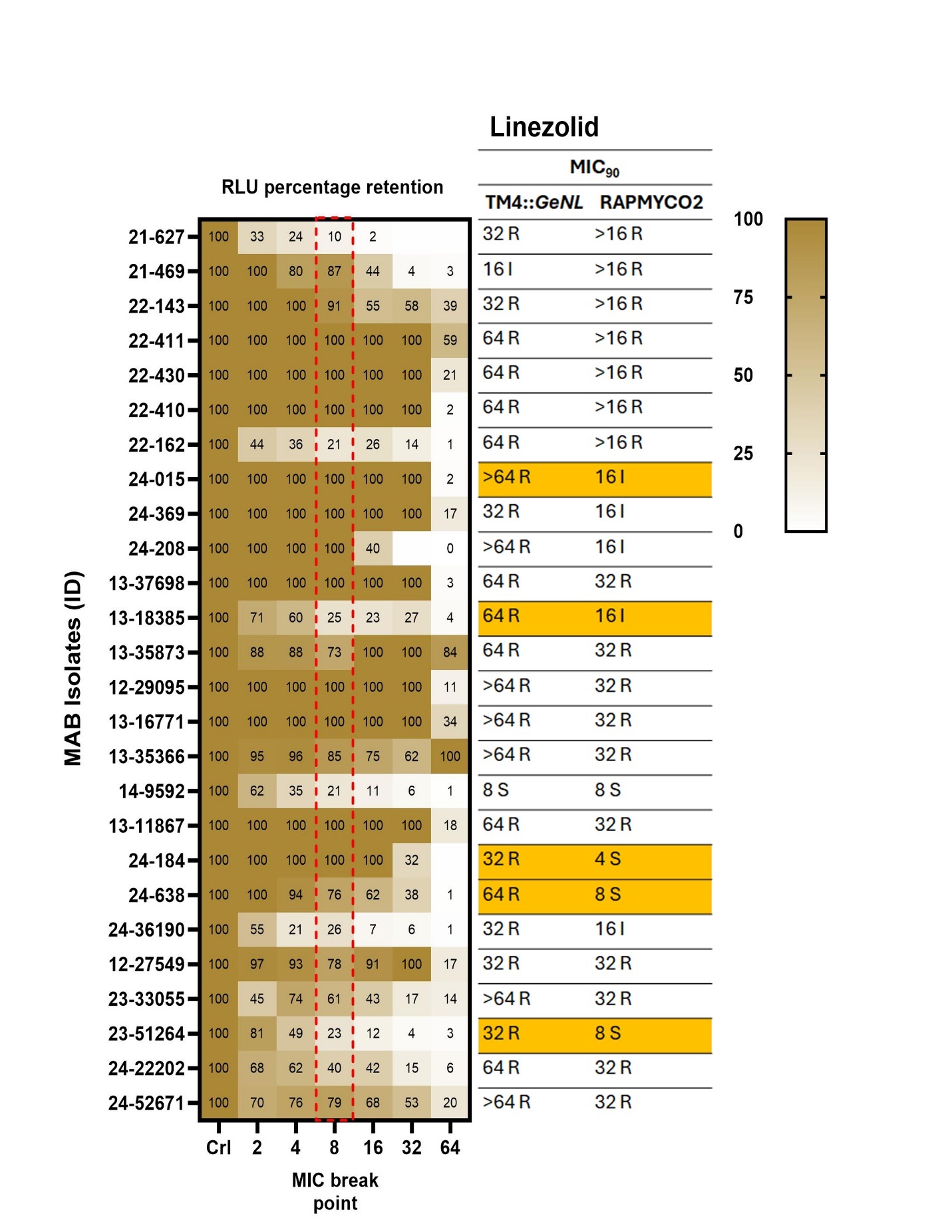


**Figure S6. Optimization of *M. abscessus* LRM DST using TM4::*GeNL*.**


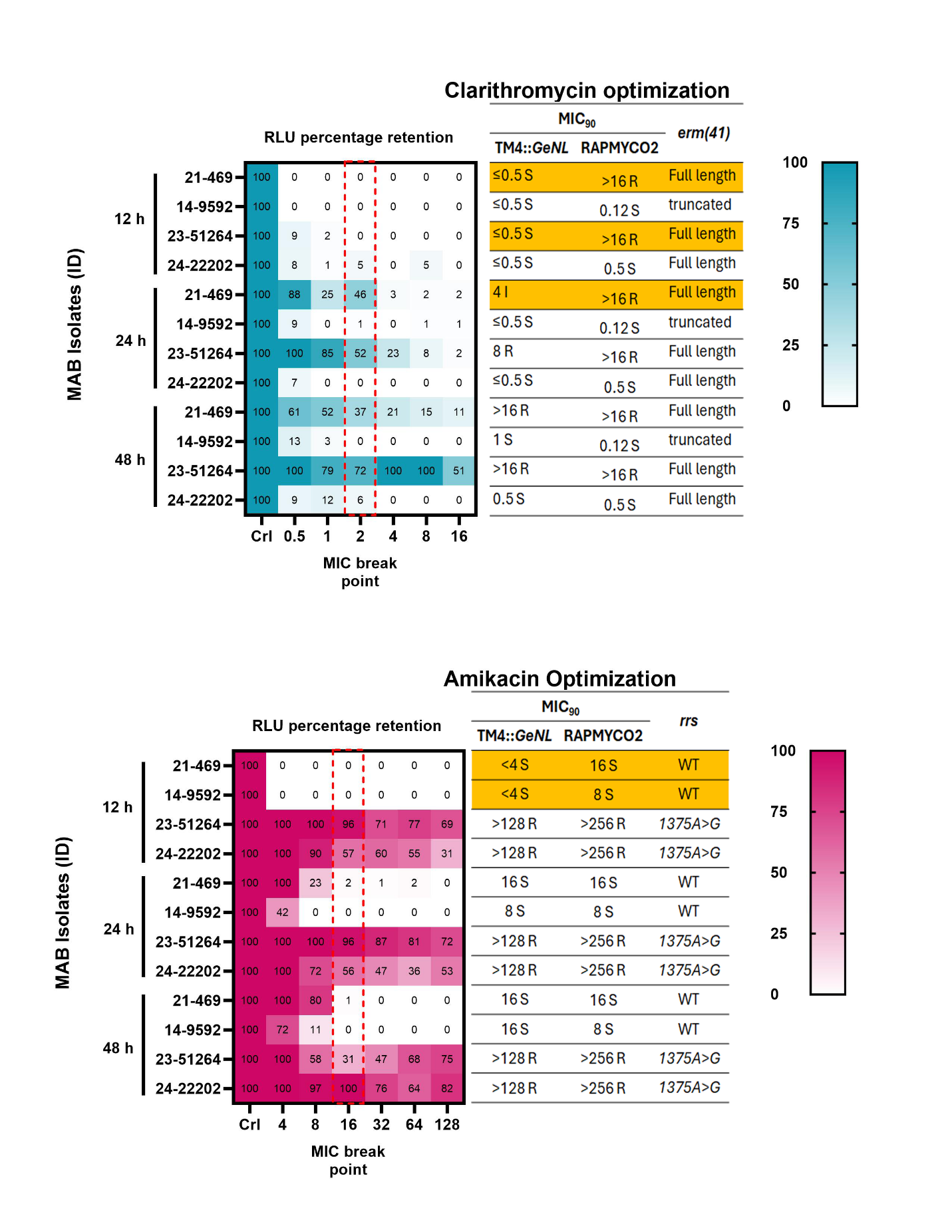

Supplement: Supplemental tables and figures — Table S1 and Fig. S1 to S6. [file jcm.00841-25-s0001.docx]
